# The Week's Work

**Published:** 1911-03-18

**Authors:** 


### The Week's Work.

### GREAT ORMOND STREET HOSPITAL.

On a subsequent page is printed a condensed report of the proceedings at a special Court of Governors of this hospital, convened to consider the recent attacks made on the hospital in the weekly press. It will be remembered that in our issue of November 12, 1910, p. 185, we dealt with the first of these attacks; and it gives us peculiar pleasure to notice that the report now published confirms all that we then advanced in deprecation of the course taken by the managers of the weekly publication referred to. The further sequence of events after the date just mentioned is instructive. Feeling, not unnaturally, that the treatment accorded to the hospital was grossly unfair, the Governors made no reply to the reckless charges made against their staff and themselves. This contemptuous silence stung the accuser to fury and resulted in further crops of "horror" as garbled, inaccurate, and sensational as the first. The silence of the authorities was not due, as the report points out, to any indifference or callousness on the part of those attacked, but rather to a determination not to submit their honour to a self-constituted tribunal whose impartiality they had such grave reason to doubt. As a matter of fact, the degree of suffering which was caused among the staff—more especially the nursing staff—by the odious charges levelled against them was very great indeed. Rather than play into the hands of unscrupulous sensation-mongers, the Governors wisely decided to conduct a searching investigation into the subject matter of the accusations; and then to publish it for all to read. This has been done, and if it is too much to hope that the sensation-mongering press will abandon its methods, still at the bar of public opinion it stands arraigned of crimes far worse than any it has laid with such levity and injustice at the door of the Great Ormond Street Hospital. On their dignified bearing under serious and long continued provocation the authorities of the hospital are to be congratulated, as well as upon their complete refutation of the slanderers who attacked them. They have also the material consolation of knowing that one of the most munificent bequests that any hospital has received of recent years was bequeathed to them *after* the publication of the attacks in question. So that on all grounds the Governors, the honorary and resident staffs, the nursing staff, and everyone connected with this splendid institution may be well content with the results of the investigation.

### MRS. BATEMAN'S WILL.

The laziness of human nature and the dangers which attend it in the case of people with money to spare was glaringly illustrated in the Chancery Division of the High Court a few days ago, when the claims of various hospitals to benefit by the will of the late Mrs. Bateman were considered. According to her will Mrs. Bateman's residuary estate was "to be divided equally between" some half-dozen institutions, one of which was described as "The Royal Hospital for Women." There is no "Royal Hospital for Women," consequently six institutions claimed to be the hospital which Mrs. Bateman had in mind. Their claims were natural and proper, because the incorrect vagueness of the designation in the will gave to each a plausible argument in its favour. The case was complicated by an affidavit which the solicitor who prepared the will had filed, and in which he recalled a conversation with the testatrix when a certain hospital for women had been recommended by him as a proper legatee. It was said that she approved and that a will was made containing this bequest. As stated, it turned out that this was not the correct name, and this parole evidence was not admitted by the judge, who directed that a scheme for the application of the money should be settled in chambers. Hospitals may be excused for grumbling at the inaccuracy of the testatrix and her adviser, for, though we should be the last people in the world to discourage legators to hospitals, generous impulses which involve half-a-dozen charities in litigation we are better without.

### THIS WEEK'S DRUG MARKET.

A moderate business has been done in the drug market during the week, but few articles are attracting special attention at the moment. Unfavourable reports continue to come from Norway concerning the cod fishing, and so far this season the yield of cod liver oil is only about half the yield at the same date last season; consequently the high quotations are maintained. Opium is unaltered in value, but at present there is no indication of a further advance, and if anything the tendency is in the other direction. There is a good demand for jalap at dearer rates. Menthol is cheaper and the market is quiet. Coca leaves are rather dearer and a slight advance in the price of santonin has been announced. There is a good demand for oil of aniseed at full prices. A fair business has been done in potassium bromide and a further advance in price should not come as a surprise. Gentian root tends somewhat dearer. Camphor is quiet. An advance in the price of cocaine hydrochloride is not improbable, and it is hinted that quotations for chloral hydrate may be raised. Ergot of rye continues scarce and dear.

